# The patterns and occupational distribution of hormonal abnormalities among men investigated for infertility in some centers in the southwest, Nigeria

**Published:** 2021-03-12

**Authors:** Muyiwa Adeleye Moronkeji, Mathias Abiodun Emokpae, Timothy Ayodele Ojo, Ruth Efe Moronkeji, Lawrence Tayo Ogundoju

**Affiliations:** ^1^Department of Medical Laboratory Science, School Of Basic Medical Sciences, University of Benin, Benin City, Edo State, Nigeria; ^2^Department of Chemical Pathology, Ladoke Akintola University of Science and Technology Teaching Hospital, Osogbo, Osun State, Nigeria; ^3^Department of Medical Microbiology and Parasitology, Ladoke Akintola University of Science and Technology Teaching Hospital, Osogbo, Osun State, Nigeria; ^4^Department of Medical Laboratory Services (Microbiology and parasitology), State Specialist Hospital, Hospital Management Board, Asubiaro, Osogbo, Osun State, Nigeria; ^5^Department of Medical Laboratory Services, University Health Centre, Federal University of Technology Akure, Akure, Ondo State, Nigeria

**Keywords:** gonadotropin-releasing hormone, follicle-stimulating hormone, luteinizing hormone, testosterone, infertility

## Abstract

**Background and aim:**

Male factor infertility results from dysfunction at various levels of spermatogenesis, sex hormone abnormalities, and occupation or workplace exposure to toxins are involved. This study was designed to determine the frequency of occupational distribution of men who were evaluated for infertility, the patterns of hormonal abnormalities, and to associate hormonal abnormalities with occupational categories in some centers in Osun State, Nigeria.

**Methods:**

Semen and 5 mL of whole blood were collected from the infertile men (n=319) who were referred to the laboratories for fertility investigation after physical and medical examination. Semen analysis was performed microscopically according to the World Health Organization manual while serum gonadotrophin-releasing hormone (GnRH), follicle-stimulating hormone (FSH), luteinizing hormone (LH), testosterone, and prolactin were determined by Enzyme-linked Immunosorbent assay technique using reagents supplied by Biorex Diagnostics Limited, Antrim, United Kingdom. The subjects were grouped based on semen characteristics.

**Results:**

More than half 164 (51.4%) of the subjects were within the age group of 31-40 years, followed by 70 (21.9%) in the age group of 21-30 years, while 67 (21.0%) was in the age group of 41-50 years. Some 133/319 (41.7%) of the subjects had normal sex hormone levels while 186/319 (58.3%) had abnormal hormone levels. The patterns of hormonal abnormalities observed were 96/186 (51.6%) normogonadotrophin-hypogonadism, 49/186 (26.3%) normogonadotrophin-hypergonadism, 14/186 (7.5%) elevated FSH levels, 15/186 (8.1%) elevated LH levels, 07/186 (3.8%) hypergonadotropic-hypergonadism, and 05/186 (2.7%) hyperprolactinemia. Artisans (OR 1.2252 95%CI 0.367-2.472), workers in chemical related industries (OR 1.667, 95%CI 0.594-4.676), and businessmen (OR 1.200, 95%CI 0.110-3.49) are more likely to be predisposed to hormonal abnormalities.

**Conclusion:**

The patterns of hormone abnormalities as well as their relative proportions are slightly different from those reported previously. Some occupations may predispose workers to hormonal disorder than the others.

**Relevance for patients:**

This is a cross-sectional study of males investigated for infertility; the contribution and patterns of hormonal abnormalities were evaluated. The possible association between workplace and infertility that may assist in the management of patients with male infertility was evaluated.

## 1. Introduction

Infertility is a disease of reproductive systems characterized by the inability of a sexually active, non-contraception couple to achieve pregnancy in 1 year after regular unprotected intercourse [1]. Globally, about 15% of all sexually active couples of reproductive age are affected by infertility and about 40-50% are attributed to abnormalities in males, called male factor infertility [2]. Infertility is not only a medical challenge; it also results in psychosocial problems such as marital disharmony, stigmatization, depression, and psychiatric challenges. Not until recently, Nigerian-sociocultural believed that infertility was attributed only to women. It is now clear that the problems are shared equally between males and females. There appears to be an increasing willingness of men nowadays than before to present for evaluation for infertility.

Male infertility ensues as a result of diminished bioavailability and dysfunctions at various levels along the hypothalamic pituitary-Gonadal axis (HPGA): Pre-testicular (damage at the hypothalamus or pituitary level), testicular (failure of the testis), post-testicular (normal testicular function but with obstruction or inflammation that leads to infertility) or a combination of these [3]. It was estimated that about 187 million couples worldwide or one out of every six couples of reproductive age are affected [4]. However, in sub-Saharan Africa, the prevalence of male infertility is higher and is currently reported to be between 30 and 50% [5-8].

The functionalities of the male reproductive system are largely dependent on hormones that are produced by endocrine glands. Abnormalities in hormone production and transportation challenges to the sites of action have been reported as one of the causes of male infertility and hormonal therapy has been used to correct the abnormality [9,10]. Furthermore, male factor fertility depends on viable production of normal quality, and quantity of healthy sperms in the testes, which, in turn, is dependent on adequate production and release of gonadotropin hormones by the pituitary gland at the base of the brain.

The etiological factors of male infertility are many and the contribution of endocrine abnormalities has been evaluated by several authors [7,8,11-14]. Endocrine involvements in male infertility are increasingly identified and the patterns vary from one region of Nigeria to another probably due to differences in lifestyle behaviors such as nutrition [15,16]. Others have attributed this to differences in environmental pollutions as a result of crude oil exploration and exploitation [17,18]. These lifestyle habits are modifiable and evolving; therefore, it is important to periodically investigate hormonal patterns of male infertility.

Available evidence indicates that physical and chemical hazards in the workplace can impact negatively on male fertility, but the workers may be unaware of such challenges. The association of industrial workers with poor indices of semen among men underdoing fertility treatment has been reported. Exposure to occupational hazards can alter the sex drive of men and women, as well as impairment of fertilization potentials. Disruption of spermatogenesis may occur with occupational exposure to organic solvents which are commonly found in industrial companies [19]. It is imperative to have a recall of work-related toxins exposure when evaluating men for infertility.

This present study was designed to determine the frequency of occupational distribution of men who were evaluated for infertility, the patterns of hormonal abnormalities, and to associate hormonal abnormalities with occupational categories in some centers in Osun State, Nigeria.

## 2. Materials and Methods

This study was conducted at some public and private medical facilities in Osun State, southwest, Nigeria, between February 2017 and July 2019. The centers include Lautech Teaching Hospital Complex, Ile-Ife, State Specialist Hospital, Osogbo, Union Diagnostic Center, Osogbo, Frangrance of Life Medical Diagnostic and Research Laboratory Services, Prime, and Seventh Day Adventist Hospital, Ile – Ife, all in Osun State. The subjects were referred to the laboratories as part of their investigations for infertility.

### 2.1. Ethical consideration

The study protocol was reviewed and approved by the Health Research Ethics Committee of Osun State Ministry of Health, Abere, Osogbo, Osun State (Ref. OSHREC/PRS/569/149) dated November 30, 2017. All study participants gave informed consent to participate in the study before they were enlisted.

### 2.2. Inclusion criteria

Thorough physical and medical examinations were conducted on the participants by the attending physicians. Only those who met the inclusion criteria were recruited in the study. They consist of males aged 21-60 years who were referred to the laboratory for semen analyses as part of their investigation for infertility, gave consent, without physical abnormalities or chronic illnesses were included in the study. Subjects without chronic clinical illnesses and had their babies within the last 1 year, whose seminal fluid concentrations were over 15 million sperm cells per milliliter according to the WHO criteria [20] were included and used as controls.

### 2.3. Exclusion criteria

After physical and clinical examinations, individuals with known pathological or congenital conditions such as severe hypertension, diabetes mellitus, sexually transmitted diseases, testicular varicocele, and genital warts were excluded from the study. Besides, individuals currently on antioxidant food supplements, smoke cigarettes, and consume alcohol were also excluded due to their high seminal reactive oxygen species levels and possibly low antioxidant activity which might lead to decreased motility and abnormal sperm morphology.

### 2.4. Sample size

The sample size (n) was calculated using an estimated prevalence of 40% from a previous study on male infertility in Nigeria (Emokpae *et al.*, 2007) and sample size determination formula by Lwange and Lemeshow [21], N=Z^2^(1-P)P/d^2^. The calculated sample size was 369 which was increased to 400 for the purpose of this study. Some 81 respondents were later excluded because they admitted to smoking and consume alcohol and 319 subjects were eventually used in the study.

### 2.5. Specimen collection

A total of 319 male subjects evaluated for fertility were included in the study, and 100 age-matched healthy men who had fathered a child within the last 12 months were recruited as controls. Semi structured questionnaire was used to collect socio-demographic data of both infertile and control subjects. The questionnaire was administered by trained research assistants at the various centers. Thereafter, the subjects were taught on how to collect semen specimen after at least 3 days of sexual abstinence and brought to the laboratory immediately. The specimens were collected in a wide-mouth container without the use of condoms or spermicidal cream. Exactly 5 mL of blood specimen was obtained from the subjects in the morning for hormonal determination. The blood was allowed to clot and was centrifuged after clot retraction at 3000 rpm for 10 min. The serum was then kept frozen at −20°C until analyses were done. The semen analyses were done immediately the specimen was received in the laboratory.

### 2.6. Laboratory analyses

#### 2.6.1. Semen analysis

For each semen sample, the color, pH, viscosity, and liquefaction time were recorded. After, liquefaction, the sperm concentration was counted in million/mL using a Mackler counting chamber (semen Analysis Chamber, ISO 9001:2000) and was viewed under a binocular microscope (Olympus) at ×100. The motility, morphology, and progressive motility were also assessed microscopically according to the WHO recommendation [20]. An appropriate quality control measure was observed as recommended by the WHO. The semen assay was done on two different occasions at 3 months interval for those semen specimens that gave abnormal results and the mean was determined and used for the results.

#### 2.6.2. Hormonal evaluations

The GnRH, FSH, LH, testosterone, and prolactin evaluations were done by Enzyme-linked-Immunosorbent Assay technique (ELISA) using reagents supplied obtained from Biorex Diagnostics Limited (ISO: 13485) Antrim, UK. The analyses were done by following the manufacturer’s protocol and read with the aid of a microplate reader. The hormones analyzed include GnRH, FSH, LH, and testosterone. The assays were based on competitive ELISA technique.

#### 2.6.3. Principle

The assay employs high affinity and specificity antibodies (enzyme and immobilized), with different and distinct epitope recognition in excess, of native antigen. On mixing monoclonal biotinylated antibody, the enzyme-labeled antibody and a serum containing the native antigen reacts, to form an insoluble sandwich complex. Simultaneously, the complex is deposited to the well through the high-affinity reaction of streptavidin and biotinylated antibody. The antibody-bound fraction is separated from unbound antigen by decantation or aspiration. The enzyme activity in the antibody-bound fraction is directly proportional to the native antigen concentration. Several different serum references of known antigen values were used to generate a dose response curve from which the antigen concentration of an unknown was calculated.

### 2.7. Statistical analysis

The data generated from the study were compared between the groups using unpaired Students-test and Chi-square. The statistical significance level was set at *P*<0.05. The statistical software SPSS version 21 (SPSS INC, Chicago, IL, USA) was used for the analysis.

## 3. Results

The findings from this study are presented in Tables 1-7. Table 1 shows the demographic characteristics of the studied participants. Most of them 164/319 (51.4%) belong to the age group 31-40 years, and the least 18/319 (5.6%) belong to age group 51-60 years. Majority of the respondents 217/319 (68%) had tertiary education while only 20/319 (6.3%) had primary level education and none was uneducated. Furthermore, 200/319 (62.7%) had no children while 119/319 (37.3%) had one or more children. About 294/319 (92.2%) had one wife while 25/319 (7.8%) had more than one wife. The occupational distribution shows that 54/319 (16.9%) were artisans, 136/319 (42.6%) were civil servants, 20/319 (6.3%) farmers, 23/319 (5.4%) were work in chemical related industries, and 86/319 (27%) were businessmen. The duration of infertility range from 2 years to 18 years; 202/319 (63.3%) were ≤5 years, 75/319(23.5%) 6-10 years, 22/319(6.9%) 11-15 years, and 20/319 (6.3%) ≥16 years. The Chi-square goodness-of-fit test between the measured variables was significant.

**Table 1:** Demographic characteristics of the studied participants

Variable	Frequency	Percentage	X^2^	*P*-value
Age group (years)				
21-30	70	21.9		
31-40	164	51.4		
41-50	67	21.0	17.52	0.005
51-60	18	5.6		
Education				
Primary	20	6.3		
Secondary	82	25.7	25.02	0.001
Tertiary	217	68.0		
No of children				
0	200	62.7		
1	78	24.5		
2	19	6.0	17.21	0.005
>3	22	6.9		
No of wife				
1	294	92.2		
2	20	6.3	13.82	0.005
3	5	1.6		
Occupation				
Artisan	54	16.9		
Civil servants	136	42.6		
Farmer	20	6.3	22.01	0.001
Chemical related job	23	5.8		
Business men	86	27.0		
Age of marriage				
≤5 years	202	63.3		
6-10 years	75	23.5	23.20	0.001
11-15 years	22	6.9		
≥16 years	20	6.3		

**Table 2:** Lifestyle characteristics and mode of semen collection

Life style	Frequency	Percentage
Regular exercise		
Yes	109	34.2
No	210	65.8
		*P<*0.001
Smokers		
Yes	0.0	0.0
No	319	100.0
Consume alcohol		
Yes	0.0	0.0
No	319	100.0
Contraceptive		
Yes	08	2.5
No	311	97.5
Coitus		
Once a week	68	21.3
Twice a week	101	31.7
Thrice a week	105	32.9
Four times a week	45	14.1
Mode of collection		
Masturbation	251	78.7
Coitus	68	21.3

**Table 3:** Occupational distribution on the subjects based on sperm characteristics

Occupation	Azoospermia: No sperm cells	Oligospermia: <15 million sperm cells/mL	Normozoospermia: >15 million sperm cells/mL	Total	Pearson’s χ^2^	*P-*value
Farmer (%)	1 (5.0)	5 (25.0)	14 (70.0)	20 (100.0)	9.19	0.316
Artisan (%)	10 (18.5)	15 (27.8)	29 (53.7)	54 (100.0)		
Civil servant (%)	10 (7.4)	83 (61.0)	43 (31.6)	136 (100.0)		
Business (%)	7 (8.1)	30 (34.9)	49 (57.0)	86 (100.0)		
Chemical related job (%)	2 (8.7)	5 (21.7)	16 (69.6)	23 (100.0)		
Total (%)	30 (9.4)	138 (43.3)	151 (47.3)	319 (100.0)		

**Table 4:** Comparison of some sex hormone levels between infertile and fertile males (mean±SD)

Sex hormones	Infertile males (*n*=319)	Fertile controls (*n=*100*)*	*P*-value
GnRH (5.0-16.0 pg/mL)	18.01±15.89	12.20±2.96	0.001
FSH (1.0-14.0 mIu/mL)	5.56±0.24	7.63±2.97	0.001
LH (0.7-7.4 mIu/mL)	5.62±2.83	3.86±1.59	0.001
TESTO. (2.5-10.0 ng/mL)	4.72±0.19	5.18±0.24	0.001

GnRH: Gonadotropin releasing hormone; FSH: Follicle stimulating hormone; Testos: Testosterone LH: Luteinizing hormone; *Significant difference

**Table 5:** Frequency and percentage distributions of sex hormone status classified as low, normal, and elevated based on the reference ranges among infertile males

Parameters	Low (%)	Normal (%)	Elevated (%)
Testosterone (2.8-10 ng/mL)	96 (30.1)	193 (60.5)	30 (9.4)
Follicle stimulating hormone (1.0-14.0 mIU/mL)	2 (0.6)	300 (94.0)	17 (5.3)
Luteinizing hormone (0.7-7.4 mIU/mL)	5 (1.57)	300 (94)	14 (4.4)
Gonadotropin releasing hormone (5.0-16.0 pg/mL)	0.0 (0.0)	304 (95.3)	15 (4.7)
Prolactin (1.8-17 ng/mL)	2 (0.6)	312 (97.8)	5 (1.6)

**Table 6:** Pattern of hormonal abnormalities among men investigated for infertility

Hormonal abnormalities	GnRH (pg/mL)(5.0-16.0)	LH (mIU/mL) (0.7-7.4)	FSH (mIU/mL) (1.0-14.0)	Testosterone (ng/mL) (2.8-10.0)	PRL (ng/mL) (1.8-17.0)
Hypergonadotropic hypergonadism (*n*=07)	**22.22±3.39**	**9.70±3.62**	**14.02±1.93**	9.22±1.66	2.81±0.40
Isolated LH elevation (*n*=15)	**18.71±3.26**	**8.52±2.78**	5.81±1.09	3.35±0.81	3.01±0.20
Isolated FSH elevation (*n*=14)	15.98±1.01	5.66±3.49	**17.43±2.85**	4.54±1.02	2.60±0.40
Normogonadotropic hypergonadism (*n*=49)	14.21±11.99	4.87±2.63	5.34±2.71	**10.66±1.26**	4.10±0.61
Normogonadotropic normogonadism (*n*=133)	11.33±19.09	5.65±2.92	5.24±0.35	4.80±0.28	3.81±0.21
Normogonadism hypogonadism (*n*=96)	12.49±13.67	5.51±2.79	5.18±2.67	**2.04±0.32**	3.12±0.311
Prolactinemia (*n*=5)	13.85±3.58	1.50±1.26	3.62±1.01	**12.04±3.72**	**24.4±0.30**

Values in bold letters are higher than upper limits of the reference ranges. GnRH: Gonadotrophin releasing hormone; LH: Luteinizing hormone; FSH: Follicle stimulating hormone; PRL: Prolactin.

**Table 7:** Abnormal hormone distribution based on occupation and their association

Occupation	Number of subjects evaluated	Subjects with abnormal hormone levels	Percentage	Unadjusted Odd Ratio (Confidence Interval)
Artisans	54	37	68.5	1.252 (0.367-2.472)
Civil servants	136	83	61.0	0.918 (0.376-2.241)
Farmers	20	08	40.0	0.648 (0.177-2.360)
Chemical related workers	23	10	43.5	1.667 (0.594-4.676)
Businessmen	86	48	55.8	1.200 (0.110-3.49)
Total	319	186	58.3	-

Odds Ratio<1 indicates that occupation has less effect, but when it is≥1, the occupation has greater effect on hormone levels

Table 2 indicates the lifestyle characteristics of the subjects; they were non-smokers, non-alcohol consumers and only 109/319 (34.2%) engage in regular exercise while majority 210/319 (65.8%) do not exercise regularly. Bivariate analysis of categorical data on exercise indicates a significant difference between those on regular exercise and those not exercising (*P*<0.001). Greater proportion of the respondents 311/319 (97.5%) do not use any form of contraceptive while 08/319 (2.5%) use one form of contraceptive.

The occupational distribution of infertile men according to sperm cell concentrations shows that 25/54 (46.3%) were artisans, 93/136 (68.4%) were civil servants, 37/86 (43.0%) were businessmen, 06/20 (30%) were farmers, and 07/23 (30.4%) men working in chemical-related companies were either azoospermia or oligozoospermia. The majority of the respondents were civil servants 136/319 (42.6%) and the least were people working in chemical-related companies 23/319 (5.8%) (Table 3).

Table 4 shows the comparison of measured sex hormones between infertile and control subjects. Serum FSH and testosterone were significantly lower (*P*<0.001) while GnRH and LH were significantly higher (*P*<0.001) in infertile than controls.

Table 5 shows the frequency of low, normal, and elevated hormone levels based on the reference ranges. It indicates that 105/186 (56.5%) had low hormone levels while 81/186 (43.5%) had elevated hormone levels among the subjects. Over-all, 186/319 (58.3%) had hormonal abnormalities while 133/319 (41.7%) had normal hormone levels.

The patterns of hormonal abnormalities observed were 96/186(51.6%) normogonadotrophin-hypogonadism, 49/186 (26.3%) normogonadotrophin-hypergonadism, 14/186 (7.5%) elevated FSH levels, 15/186 (8.1%) elevated LH levels, 07/186(3.8%) hypergonadotropic-hypergonadism, and 05/186 (2.7%) hyperprolactinemia (Table 6).

Table 7 shows the occupational distribution of respondents with hormonal abnormalities and their possible association. Individuals who are artisans (OR 1.2252 95%CI 0.367-2.472), workers in chemical related industries (OR 1.667, 95%CI 0.594-4.676), and businessmen (OR 1.200, 95%CI 0.110-3.49) are more likely to be predisposed to hormonal abnormalities.

There was no statistically significant association between the occupation of the test subjects and the subtype of infertility with (X)^2^ (8)= 9.391 and *P*>0.05.

## 4. Discussion

Infertility is a major public health concern among couples, and occupational hazards may be the main cause of infertility in men. Several authors have reported that physical and chemical hazards in the workplace can have an adverse effect on male fertility [22]. Occupation and exposure to harmful environmental contaminants are well known endocrine disruptors and are leading causes of infertility. Preventing adverse occupational effects on male infertility is important for health-care providers. Therefore, the provision of employee awareness, providing appropriate preventive measures when performing hazardous jobs would go a long way to preventing infertility. Furthermore, understanding the functions of the hypothalamic-pituitary-gonadal (HPG) axis is important in the investigation and management of male infertility. Despite the high control mechanisms involved in spermatogenesis, any derangement within the axis can result in the male reproductive failure.

In this study, 58.3% of men investigated for infertility had hormonal abnormalities which is higher than 46.9% reported from Ilorin [14], 40% among azoospermia, 40% among oligozoospermia from Kano, respectively [7,12], and 7.3% reported from Maiduguri [23], but lower than 80.1% among males investigated for infertility reported from Enugu [8]. Male infertility of endocrine origin can manifest in several patterns. The observation of significantly lower FSH, testosterone, and higher LH levels aligns with the previous studies [24-26], but at variance with other authors [14,27]. The observed lower FSH and higher LH and testosterone levels may be attributed to testicular damage not involving the Leydig cells, but involves in androgen biosynthesis or extra-gonadal androgen synthesis [14].

The patterns of hormonal abnormalities differ from place to place. Four patterns (17.1% hypergonadotrophin-hypogonadism, 15.7% hypogonadotrophin-hypogonadism, 5.7% isolated decreased FSH and increased testosterone, and 1.4% hyperprolactinemia) were reported from Kano [12]. In the same vein, five different patterns of hormonal profile abnormalities were reported among men investigated for infertility in Ilorin; (1.5% hypogonadotropic- hypogonadism, 11.5% hypergonadotropic-hypogonadism, 17.7% hypergonadotropic-normogonadism, 16.2% normogonadotropic-hypogonadism, and 53.1% normogonadotropic normogonadism) [14]. Six patterns of hormonal abnormalities were however observed in this study among infertile men with abnormal hormone levels, which are; 51.6% (96/186) normogonadotrophic-hypogonadism, 26.3% (49/186 normogonadotrophic-hypergonadism, 7.5%(14/186 elevated FSH, 8.1% (15/186) elevated LH levels, 3.8% (07/186) hypergonadotropic-hypergonadism, and 2.7% (05/186) hyperprolactinemia. The observation of 51.6% males with biochemical features suggestive of primary testicular insufficiency aligns with the previous studies where 41.7% and 54.3%, respectively, were reported among infertile males who had biochemical features indicating primary testicular failure [8,23]. Such findings may be an indication of seminiferous tubular damage which may involve the Leydig cells. Individuals with this condition may benefit from sperm retrieval and *in vitro* cytoplasmic sperm injection techniques to achieve conception [14].

The observed isolated elevation of LH and FSH levels may be due to attempts to stimulate the Sertoli cells and Leydig cells, respectively, to produce androgen proteins and testosterone that is necessary for spermatogenesis. The high FSH level may also be an indication of decreased testicular function which can be attributed to altering feedback mechanism along the HPG axis.

The 2.7% hyperprolactinemia observed in this study is lower than 5.1% reported by Ozoemena *et al*. [8], 15.4% reported by Oladosu *et al*. [14], 11% by Masud *et al*. [28], and 12.2% by Soler-Fernadez *et al*. [29]. High serum prolactin level hurts spermatogenesis and steroidogenesis through its action on prolactin receptors on Sertoli cells and Leydig cells in the testes to cause hypogonadism by altering the pulsatile release of gonadotrophins [28]. Although hyperprolactinemia is not common among men with infertility, subjects with hyperprolactinemia may be investigated for thyroid dysfunction. About 4.7% of individuals were observed to have high levels of GnRH levels in this study. This is consistent with the previous study, where it was reported that acquired defects in GnRH release commonly referred to as the GnRH pulse generator [30]. It was commonly observed in women as a result of stress, excessive exercise, and eating disorders that impair reproductive potentials. Some authors have reported acute reversible changes in GnRH-induced gonadotrophin secretion among men with severe stress and illness [12].

Evidence linking work-place or occupational exposure to harmful effects on male infertility is on the increase. Infertile men might benefit from assessment of work-place contribution to their condition. The highest proportions of infertile men (68.5%) were artisans while the lowest 40.0% were farmers. The occupational distributions likely to be associated with abnormal hormone levels among participants are artisans (OR 1.25 95% CI 0.367-2.472), workers in chemical related industries (OR 1.667 95% CI 0.594-4.676), and businessmen (OR 1.200 95% CI 0.110-3.49). This is inconsistent with the previous study, where no association with occupation was observed among 1164 males treated for infertility. The authors reported no significant difference in the measured fertility indicators between occupational categories [22]. A significant decline in sperm quality and endocrine abnormalities among infertile men of different occupations has been reported [31].

In this study, overwhelming number of respondents (41.7%) had normal hormone levels. The cause of their infertility in this group is not immediately ascertained since the etiologies of male infertility are numerous and include genetic, physical abnormalities, injuries, drugs, infections of the genital tract, radiation, toxins, or unexplained [32]. The role lifestyle factors play in the etiology of infertility has been a subject of interest among researchers. Recently, some authors have provided evidence of a correlation between lifestyle behaviors and infertility in both men and women. The enumerated lifestyle factors include delayed childbearing due to pursuit of career or education, age of starting a family, eating of fat-rich diets, smoking, alcohol and caffeine consumption, exercise, risky sexual behaviors, drug misuse, anxiety/depression, cellular phones, and radiation among others [33]. In this study, majority of the respondents do not carry-out regular exercise and the difference in the number of respondents who carryout regular exercise and those who do not was statistically significant (*P*<0.001). Available evidence suggests that exercise may contribute to improvement in the quality of sperm parameters from 9.7% to 15.2% in men performing regular exercise of 1 h, at least 3 times a week [34]. Others have observed that moderate regular exercise may lead to the reduction of seminal plasma oxidative stress than rigorous regular exercise [35]. Regular moderate exercise was recommended and may improve fertility potential among infertile subjects [33].

The majority of the respondents (51.4%) were in the age group (31-40 years). This aligns with a previous study by Benbella *et al*. [36] among Moroccan subjects, where most of the infertile men were between the ages ranges of 35 and 40 years (34.8%). Variation in the age range and percentage could be due to population size differences.

## 5. Conclusion

This study has demonstrated the various patterns of endocrine abnormalities among males investigated for infertility. The patterns of hormonal abnormalities as well as their relative proportions are slightly different from those previously reported. Some occupations may predispose workers to hormonal disorders than the others.
